# Increased hemoglobin–oxygen affinity ameliorates bleomycin‐induced hypoxemia and pulmonary fibrosis

**DOI:** 10.14814/phy2.12965

**Published:** 2016-09-13

**Authors:** Xin Geng, Kobina Dufu, Athiwat Hutchaleelaha, Qing Xu, Zhe Li, Chien‐Ming Li, Mira P. Patel, Nicholas Vlahakis, Josh Lehrer‐Graiwer, Donna Oksenberg

**Affiliations:** ^1^ Global Blood Therapeutics South San Francisco California

**Keywords:** Dyspnea, hypoxia, idiopathic pulmonary fibrosis, quality of life

## Abstract

Although exertional dyspnea and worsening hypoxia are hallmark clinical features of idiopathic pulmonary fibrosis (IPF), no drug currently available could treat them. GBT1118 is a novel orally bioavailable small molecule that binds to hemoglobin and produces a concentration‐dependent left shift of the oxygen–hemoglobin dissociation curve with subsequent increase in hemoglobin–oxygen affinity and arterial oxygen loading. To assess whether pharmacological modification of hemoglobin–oxygen affinity could ameliorate hypoxemia associated with lung fibrosis, we evaluated GBT1118 in a bleomycin‐induced mouse model of hypoxemia and fibrosis. After pulmonary fibrosis and hypoxemia were induced, GBT1118 was administered for eight consecutive days. Hypoxemia was determined by monitoring arterial oxygen saturation, while the severity of pulmonary fibrosis was assessed by histopathological evaluation and determination of collagen and leukocyte levels in bronchoalveolar lavage fluid. We found that hemoglobin modification by GBT1118 had strong antihypoxemic therapeutic effects with improved arterial oxygen saturation to near normal level. Moreover, GBT1118 treatment significantly attenuated bleomycin‐induced lung fibrosis, collagen accumulation, body weight loss, and leukocyte infiltration. This study is the first to suggest the beneficial effects of hemoglobin modification in fibrotic lungs and offers a promising and novel therapeutic strategy for the treatment of hypoxemia associated with chronic fibrotic lung disorders in human, including IPF.

## Introduction

Idiopathic pulmonary fibrosis (IPF) is a chronic disease of unknown etiology that is characterized by progressive fibrotic destruction of the lung, resulting in worsening dyspnea and progressive loss of lung function (Wilson and Wynn 2009; Raghu et al. 2011). Currently, about 5 million people worldwide are affected by IPF with over 130,000 patients in the United States. The median patient survival time is approximately 4 years from the time of diagnosis (Raghu et al. 2006, 2011). Until recently, there were no approved therapies for IPF. In 2015, the antifibrotic drugs, pirfenidone and nintedanib, were approved based on demonstrating a reduction in the decline of lung function (Forced vital capacity). However, a survival advantage was not found, nor were key disease symptoms consistently clinically impacted (King et al. 2014; Richeldi et al. 2014; Harari and Caminati 2015). Therefore, there continues to be a significant need for novel and effective therapeutic drugs for IPF patients, especially to improve symptoms and quality of life (QOL).

A prominent clinical feature of IPF is progressive hypoxemia, resulting in exertional dyspnea and eventually dyspnea at rest. The recently approved drugs did not have a significant impact on oxygen saturation or dyspnea (Nishiyama et al. 2007; Parshall et al. 2012; Bodempudi et al. 2014). Dyspnea or hypoxemia induced physical activity limitation is a prominent driver of QOL impairment among IPF patients (Swigris et al. 2014). Hypoxemia caused by pulmonary fibrosis refers to oxygen deficiency in arterial blood and reduced percentage saturation of hemoglobin (Hb) with oxygen. When oxygen (O_2_) loading of Hb is compromised in the disease lungs, such as fibrotic lungs, an increase in Hb–O_2_ affinity may be of benefit by improving the loading of Hb with O_2_, consequently increasing arterial O_2_ content and O_2_ delivery to tissues. In support of this hypothesis, increased Hb–O_2_ affinity has been shown to increase survival, improve cardiovascular function, and systemic oxygenation during acute hypoxia (Eaton et al. 1974; Yalcin and Cabrales 2012). Moreover, humans or animals with high Hb–O_2_ affinity adapt better to acute and long‐term exposures to high‐altitude hypoxia than their relatives with normal Hb–O_2_ affinity (Hebbel et al. 1978; Black and Tenney 1980).

The Hb–O_2_ affinity can be modified with Hb allosteric effectors. GBT1118 is an analog of GBT440, a novel orally bioavailable small molecule that binds covalently and reversibly via Schiff base to the N‐terminal valine of the Hb alpha chain and allosterically modulates the Hb–O_2_ affinity (Oksenberg et al. 2016). It elicits a concentration‐dependent left shift in the O_2_–Hb dissociation curve with subsequent increase in Hb–O_2_ affinity and arterial O_2_ loading (Oksenberg et al. 2016). Therefore, we investigated whether pharmacologically increasing Hb–O_2_ affinity with GBT1118 could ameliorate hypoxemia associated with lung fibrosis induced by bleomycin in mice (Degryse and Lawson 2011; Moore et al. 2013). This article reports the profound antihypoxemic and potential antifibrotic effects of GBT1118 achieved via increasing Hb–O_2_ affinity. These data establish pharmacological modification of Hb–O_2_ affinity as a promising and novel therapeutic strategy for the treatment of chronic fibrotic lung disorders and pave the way for the clinical development of “first in class” molecules that treat IPF symptoms by improving hypoxemia.

## Materials and Methods

### Bleomycin model

The in‐life portion of the mouse study was performed at Aragen Biosciences (Morgan Hill, CA). Forty‐eight C57B/L6 male mice aged 7–8 weeks (Simonsen Laboratory, Gilroy, CA) were studied. Animals were administered 3 U/kg bleomycin sulfate USP (Teva Pharmaceuticals, North Wales, PA) or saline via oropharyngeal route (Walters and Kleeberger 2008). Body weights of all mice were recorded daily during the study. Animals were closely monitored on the days of dosing and daily until the end of the study.

### Administration of GBT1118

GBT1118 was synthesized at Global Blood Therapeutics and formulated in dimethylacetamide:polyethylene glycol 400 (PEG400):40% cavitron at a 1:5:4 ratio. GBT1118 (low dose: first day 50 mg/kg followed by 40 mg/kg daily; or high dose: first day 150 mg/kg followed by 85 mg/kg daily) or vehicle control was administered via oral gavage to bleomycin‐treated mice once daily from days 8 to 15 (Walters and Kleeberger 2008).

### Pharmacological analysis

The oral exposure of GBT1118 and Hb–O_2_ dissociation curve measurements were conducted at Global Blood Therapeutics (South San Francisco, CA) and determined by the methods described previously (Oksenberg et al. 2016). Samples were taken 4 h following the last dose of the chronic dosing regimen.

### Arterial blood gases and O_2_ saturation analysis

On days 7 and 14 after bleomycin or saline administration, 50 *μ*L of arterial blood from the tail artery were used for the measurement of arterial oxygen saturation (S_a_O_2_) using a whole blood co‐oximeter (GEM OPL; Instrumentation Laboratory, Bedford, MA). An additional 100 *μ*L aliquot of blood was collected from the tail artery and arterial blood gases (ABG) were measured using i‐STAT Handheld Blood Analyzer (ABBOTT, Lake Bluff, IL). For each mouse, arterial pH, pCO_2_ (partial pressure of carbon dioxide, mmHg), and pO_2_ (partial pressure of O_2_, mmHg) were measured.

### Bronchoalveolar lavage fluid analysis

Bronchoalveolar lavage fluid (BALF) was collected from the lungs of the animals by lavaging the lung with 1 mL Hank's balanced salt solution. The BALF was centrifuged and leukocytes in the cell pellet were counted using the trypan blue exclusion method. Collagen content of the BAL was determined by quantifying total soluble collagen in the BALF supernatant using the Sircol collagen dye binding assay according to the manufacturer's instructions (Biocolor Ltd., Carrick Fergus, U.K.).

### Histopathological analysis

The histopathological analysis was conducted at Seventh Wave Laboratories (Chesterfield, MO). The lung samples were processed and embedded with all lobes from each mouse in one paraffin block. Coronal sections through the four major lobes were stained with Masson's trichrome. For each animal, consecutive lung fields were examined in a raster pattern using a 20× objective lens and a 10× ocular lens (200×). A modified Ashcroft score (Hubner et al. 2008) was recorded for each field.

### Statistical analysis

All data are presented as mean (±standard error of the mean, SEM). Groups are compared by *t* test and one‐way ANOVA. Statistical comparison and graphical representations were performed using Prism 6.0 (GraphPad software, San Diego, CA). Differences were considered statistically significant if *P‐*value was less than 0.05.

## Results

### GBT1118 increases Hb affinity for O_2_ in vivo

**Figure 1:**
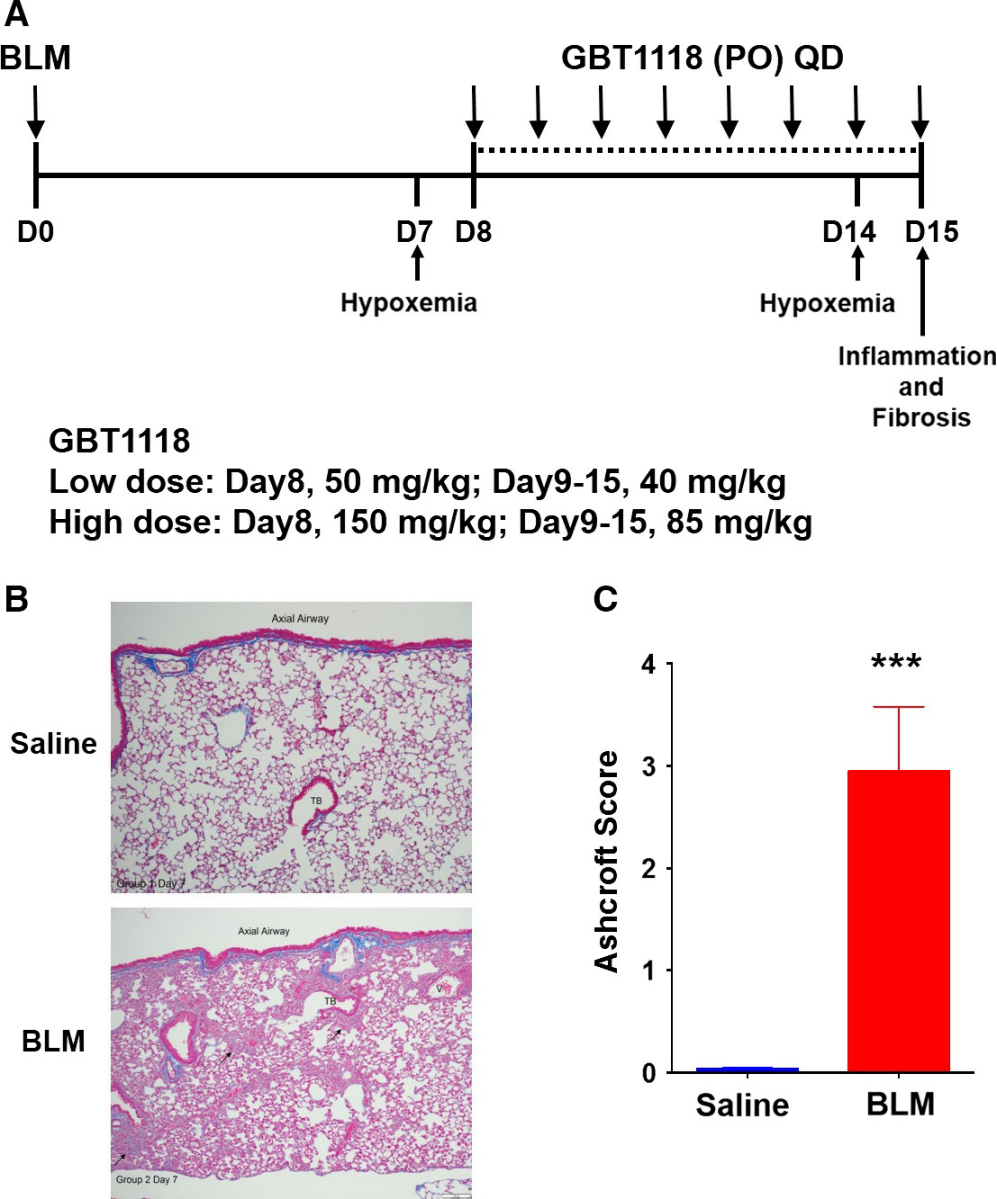
Study design. (A) GBT1118 therapeutic treatment schedule. Animals were given a single intratracheal challenge of bleomycin (BLM) or saline. Two different doses of GBT1118 or vehicle were given orally (PO) once a day (QD) from days 8 to 15. Oxygen saturation and arterial blood gases were evaluated at 7 and 14 days after challenge. Inflammation and fibrosis were accessed at day 15 after BLM. (B) Representative lung sections from day 7 mice were stained with Masson's trichrome to visualize collagen deposition (blue). (C) Ashcroft scores representing the morphologic fibrotic changes in the lungs of saline‐exposed control mice and bleomycin‐exposed day 7 mice from (B). Data represent the mean ± standard error of the mean of *n* = 5 mice per group (***P < 0.0001).

Mice were randomized into four treatment groups: high‐dose GBT1118, low‐dose GBT1118, GBT1118 vehicle, or saline. Animals were administered a single oropharyngeal dose of bleomycin (BLM, 3 U/kg) or saline on day 0. The development of fibrosis before day 8 was confirmed by histopathology (Fig. 1B, C). Subsequently from days 8 to 15 the GBT1118‐treated animals were administered one of two different dosing regimens (low dose: first day loading dose 50 mg/kg followed by maintenance dose 40 mg/kg daily; or high dose: first day 150 mg/kg followed by 85 mg/kg daily) once daily (QD) from days 8 to 15 (Fig. 1A).

**Figure 2:**
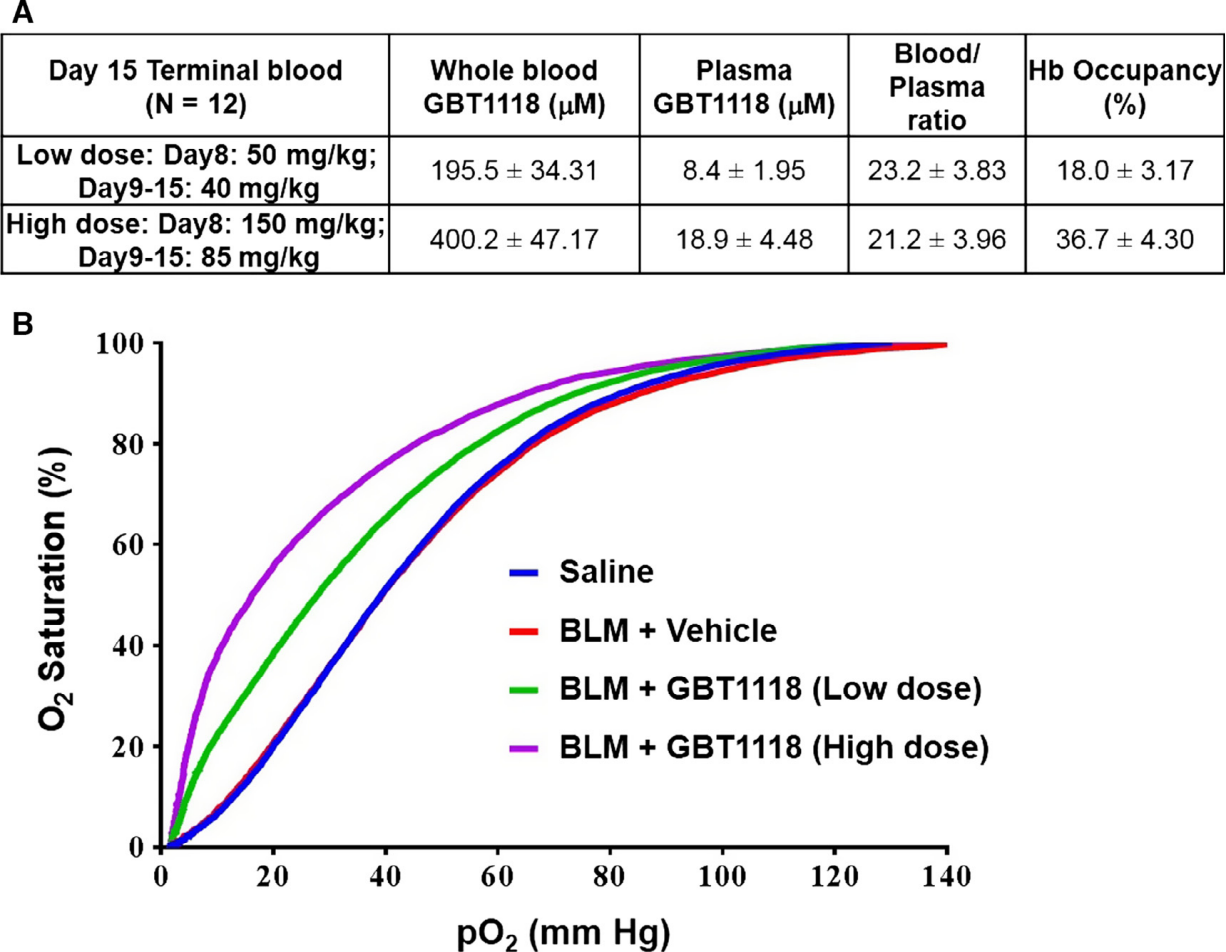
Pharmacological characterization of GBT1118 as a hemoglobin modifier in bleomycin‐treated mice. (A) Pharmacokinetics analysis. GBT1118 blood and plasma concentrations were determined by mass spectrometry 4 h after the last dose. The results shown represent the mean of *n* = 12 in each group and standard deviation. (B) Pharmacodynamic analysis. The effect of oral dosing of GBT1118 on ex vivo oxygen–hemoglobin dissociation curves in bleomycin‐treated mice. The representative oxygen–hemoglobin dissociation curves are shown (*n* = 3).

The pharmacokinetic profile of GBT1118 in bleomycin‐treated mice shows the low‐ and high‐dose regimens of GBT1118 achieved 18.0% and 36.7% of calculated Hb occupancy, respectively. Hb occupancy represents the proportion of Hb bound by GBT1118 and was calculated as the ratio of the concentration of GBT1118 to Hb in blood. GBT1118 was found to quickly partition into the red blood cells (RBCs) with a high blood/plasma ratio of approximately 22:1 which is equivalent to RBC/plasma ratio of 102:1 (Fig. 2A). This high RBC/plasma ratio indicated a preferential partitioning of GBT1118, into the RBCs and with the relatively low concentration in plasma off target toxicities should be minimized and the therapeutic index maximized.

The Hb–O_2_ dissociation curves were determined ex vivo in whole blood and correlated with the GBT1118 blood concentrations following oral dosing for 8 consecutive days in bleomycin‐treated mice. The representative Hb–O_2_ dissociation curves from GBT1118‐treated mice demonstrated a significant left shift in a dose‐dependent manner indicating a higher Hb–O_2_ binding affinity (Fig. 2B). These data indicate that oral dosing of GBT1118 increases Hb–O_2_ affinity in bleomycin‐treated mice.

### Administrations of GBT1118 rescue bleomycin‐induced hypoxemia

**Figure 3:**
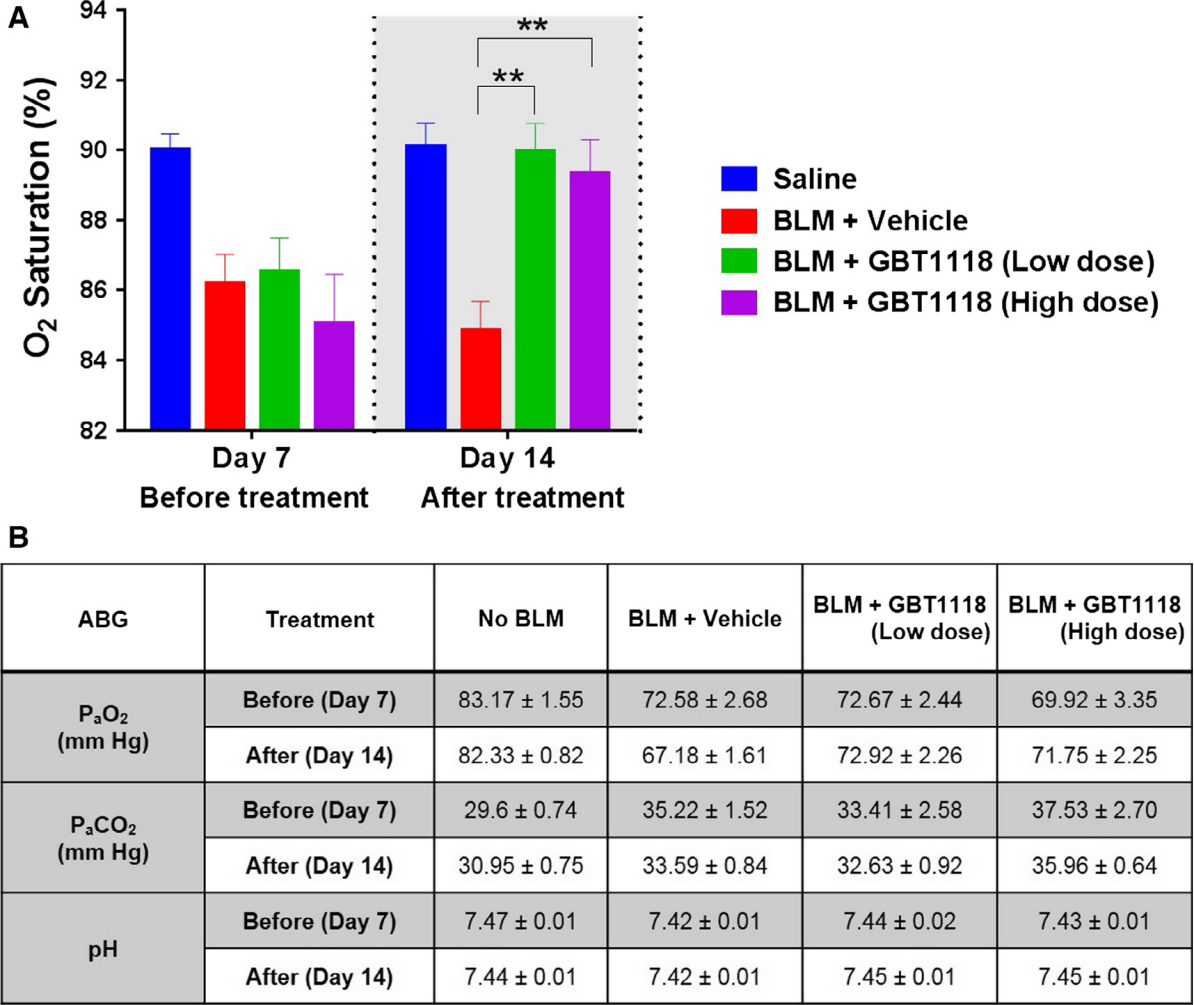
Effects of GBT1118 on bleomycin‐induced hypoxemia at days 7 and 14. (A) Arterial oxygen saturation (S_a_O_2_) and (B) the arterial blood gases (ABG) were analyzed on days 7 and 14 (before and after treatment; data are shown as means ± SEM; **P < 0.01, *n* = 12 per group).

Responses to hypoxia in mice treated with GBT1118 were first evaluated by measuring S_a_O_2_. S_a_O_2_ decreased over time in bleomycin‐treated mice. Both GBT1118‐treated groups showed a decrease in S_a_O_2_ on day 7 before GBT1118 treatment and subsequent return toward control values following treatment with GBT1118 for 7 consecutive days. In contrast, arterial oxygenation levels of vehicle‐treated mice further declined throughout the study (Fig. 3A). Please note the degree of hypoxemia was relatively mild in this bleomycin model and completely rescued by both doses of GBT1118 treatment. These findings demonstrate that GBT1118 treatment significantly reduces hypoxemia by increasing O_2_ saturation and therefore O_2_ content.

In addition, the ABG were analyzed on days 7 and 14. At day 7, in bleomycin‐treated mice, arterial O_2_ tension (P_a_O_2_) was significantly decreased, indicating an impairment of pulmonary gas exchange. In vehicle‐treated bleomycin mice, the P_a_O_2_ continued to decline until day 14. In contrast, low‐dose GBT1118 treatment prevented further decline of P_a_O_2_, indicating a beneficial effect on disease progression. In the high‐dose GBT1118 group, a trend for an increase in P_a_O_2_ was observed (Fig. 3B). No significant changes were observed on arterial carbon dioxide tension (P_a_CO_2_) and pH measurement (Fig. 3B).

### Administrations of GBT1118 ameliorate bleomycin‐induced lung fibrosis

**Figure 4:**
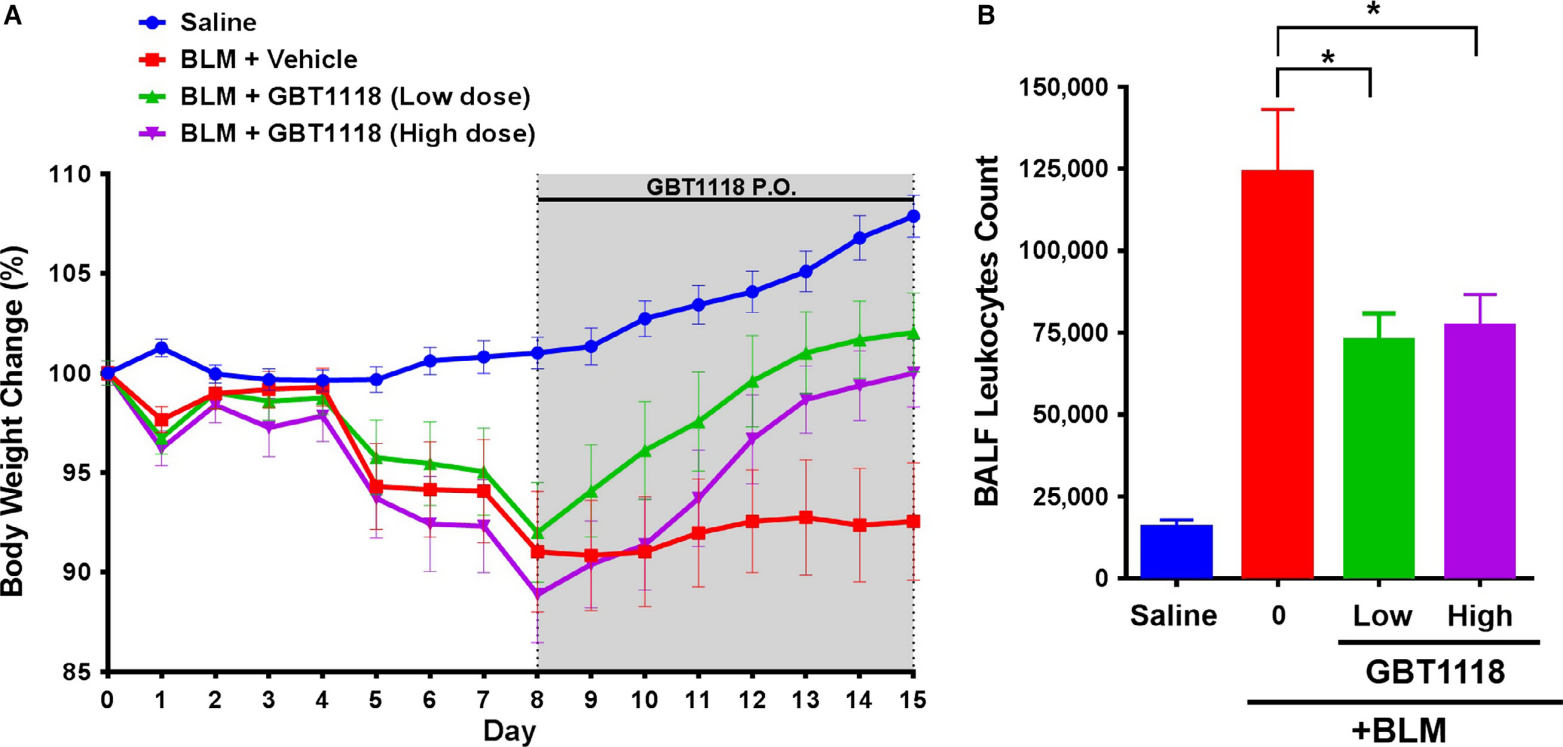
Effects of GBT1118 on bleomycin‐induced changes in body weight and leukocyte infiltration. (A) Time course of percentage changes in body weight after bleomycin (BLM) or saline treatment. Data represent the mean ± standard error of the mean of *n* = 12 mice per group. (B) Leukocyte count in bronchoalveolar lavage fluid (BALF). Data represent the mean ± standard error of the mean of *n* = 12 mice per group (*P < 0.05).

A recognized feature of bleomycin‐induced lung fibrosis in small rodents is prominent loss of body weight. In mice administered with saline, daily weight gain was observed. Animals treated with bleomycin and vehicle showed typical and persistent body weight loss after bleomycin exposure. In contrast, bleomycin challenged animals treated with either doses of GBT1118 underwent steady weight gain after the start of dosing GBT1118 on day 8, despite transient body weight loss prior to GBT1118 treatment (Fig. 4A). These data indicate that treatment with GBT1118 improves the overall health status in bleomycin‐treated animals.

In the bleomycin model, mice develop extensive pulmonary fibrosis as well as a profound leukocyte infiltration into lungs; thus, the effect of GBT1118 treatment on the alteration of pulmonary leukocyte numbers was examined. We found that treatment with GBT1118 was associated with decreased inflammation, as evidenced by a significant reduction in total leukocyte cells recovered in BALF on day 15 (Fig. 4B). This finding demonstrates that GBT1118 treatment attenuates pulmonary inflammation in this model.

**Figure 5:**
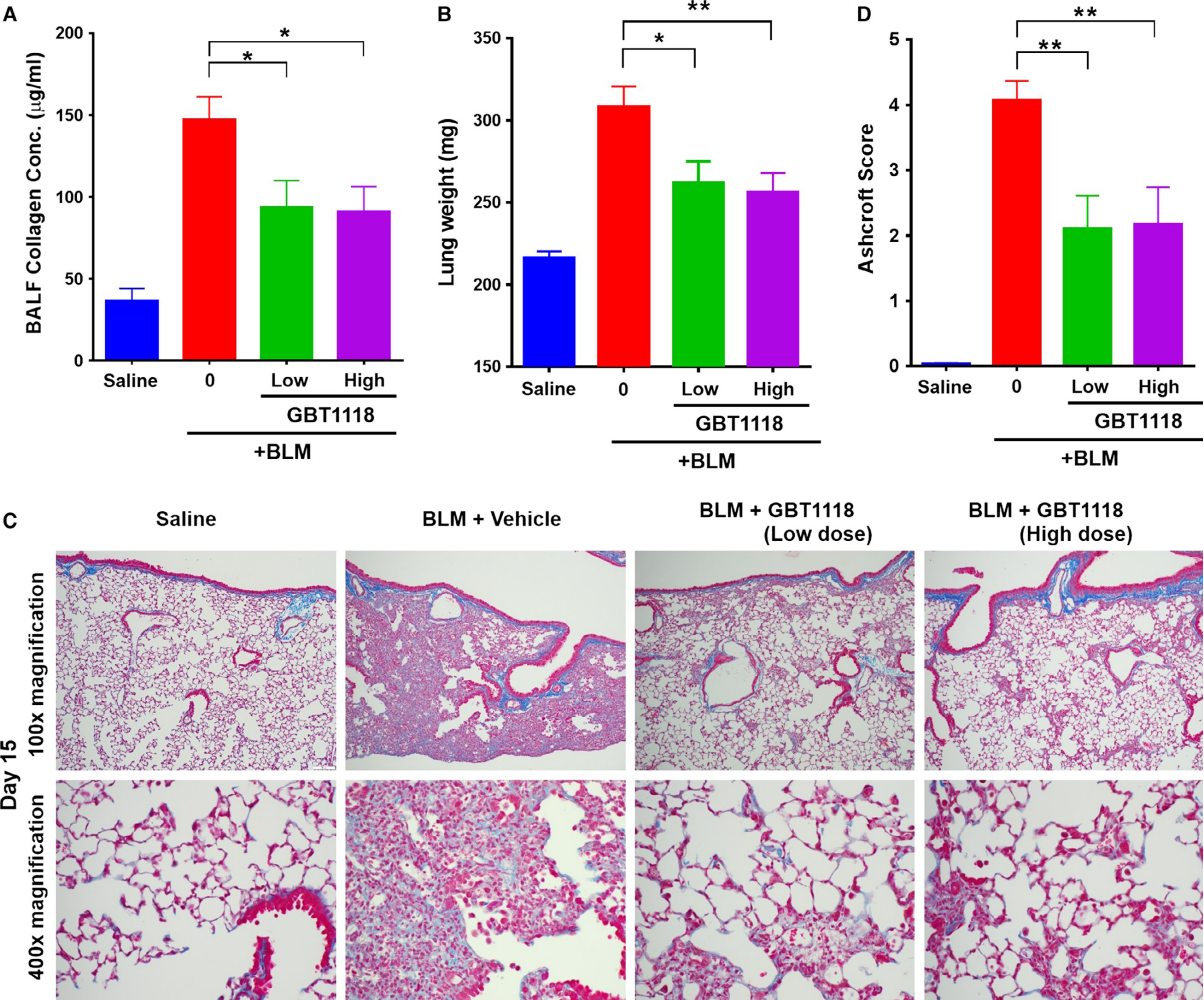
Effects of GBT1118 on bleomycin‐induced lung fibrotic lesions. Pulmonary fibrosis was induced by administering mice a single dose of bleomycin. (A) Soluble collagen in bronchoalveolar lavage fluid (BALF) on day 15 by a Sircol collagen dye binding assay. (B) Lung wet weights from mice exposed to saline or mice exposed to bleomycin and analyzed after GBT1118 or vehicle treatment from days 8 to 15. (C) Lung sections from day 15 mice were stained with Masson's trichrome to visualize collagen deposition (blue). (D) Ashcroft scores representing the morphologic fibrotic changes in the lungs of saline‐exposed control mice, and vehicle and GBT1118 treatment groups for bleomycin‐exposed mice (data are expressed as means ± SEM; *P < 0.05; **P < 0.01).

The key marker of bleomycin‐induced lung fibrosis is excessive collagen deposition. As expected, when compared to saline control, bleomycin administration led to an elevation in soluble collagen content in BALF on day 15 as assessed by a Sircol collagen dye binding assay. GBT1118 treatment resulted in a significant reduction in soluble collagen protein in the lungs (Fig. 5A). The reduction in fibrosis was confirmed by quantitative measurement of lung wet weights at necropsy. The lungs of bleomycin mice administered with vehicle control were significantly heavier than lungs from GBT1118‐treated mice, suggestive of reduced fibrotic disease in treated animals (Fig. 5B). These results indicate that GBT1118 improves pulmonary fibrosis in the bleomycin murine model.

To confirm the therapeutic effects of GBT1118 treatment on the indices of pulmonary fibrosis, lung sections from day 15 mice were stained with Masson's trichrome to visualize collagen deposition. Vehicle‐treated bleomycin lungs were fibrotic and had extensive collagen deposition, thickened pulmonary interalveolar septum, and obliteration of the alveolar airspaces by collagen. In contrast, GBT1118‐treated lungs showed diminished collagen deposition in both doses; many alveoli did not exhibit septal fibrosis and resembled the parenchyma in lungs without bleomycin exposure (Fig. 5C). Ashcroft scoring to quantify morphologic fibrosis was performed, and GBT1118 treatment improved overall scores by approximately 50% (Fig. 5D). These results suggest that GBT1118 inhibits pulmonary fibrosis in this bleomycin mouse model.

## Discussion

Suitable animal models of IPF are lacking (Roman et al. 2013) and have been identified as a research priority for the IPF field (White et al. 2016). In our attempt to elucidate the efficacy of GBT1118 drug effects were explored in the most commonly used animal model of lung fibrosis: the bleomycin‐induced model. The results from this in vivo therapeutic study provide support for the potential use in IPF of a molecule that increases Hb–O_2_ affinity. GBT1118 treatment not only restored arterial O_2_ to normal levels, but also significantly inhibited the increase in numbers of inflammatory cell infiltrates, reduced collagen in BALF, and resulted in an approximately 50% reduction in fibrosis (histopathological changes in lung tissue). Additionally, GBT1118 administration ameliorated the loss of body weight associated with bleomycin exposure.

Exertional dyspnea and worsening hypoxia associated with hypoxemia are prominent clinical features of IPF progression as fibrosis increases and ventilation–perfusion mismatch worsens. With worsening hypoxemia patient QOL for IPF patients is significantly impacted including limitation of their daily activities. The data from this study suggest that a Hb modifier that enhances Hb–O_2_ affinity and improves arterial O_2_ uploading could potentially be used to treat hypoxemia associated with pulmonary fibrosis. Although a GBT1118‐induced left shift could theoretically decrease O_2_ release from Hb, our in vitro data show that GBT1118‐modified Hb remains sensitive to the Bohr effect with intact unloading of O_2_ under low‐pH conditions, and in vivo data from different animal models of hypoxia challenge are consistent with increased O_2_ extraction and consumption by tissues but not any tissue hypoxia (Yalcin and Cabrales 2012; Cabrales et al. 2015; Oksenberg et al. 2016).

The health benefits of modifying Hb could be substantial: improvement of hypoxemia can prevent dyspnea and help patients preserve an active lifestyle and a better QOL. Moreover, it could potentially impact the associated comorbidities of IPF including pulmonary hypertension and sleep‐disordered breathing. Reduction in nocturnal desaturations and the detrimental effects of systemic hypoxemia associated with obstructive sleep apnea, which has a prevalence as high as 88% in patients with IPF (Lancaster et al. 2009; Pihtili et al. 2013), may impact the course of IPF and associated complications (Kolilekas et al. 2013; Mermigkis et al. 2015).

Interestingly, the data suggest that a Hb modifier that improves hypoxemia in this bleomycin model could potentially retard the progression of pulmonary fibrosis. A dose response of GBT1118 was not seen here, which may be due to the fact that the degree of hypoxemia was relatively mild and completely rescued by both doses (Fig. 3A). It will be necessary to inspect whether GBT1118 has direct effects on oxidant activity, inflammation, and fibrosis in the future. Despite these, the overall beneficial effects of GBT1118 were clear. The role of hypoxia driving the progressive fibrotic nature of the disease has not been fully explored previously. In aggregate, our own and previously published data might indicate a potential role for hypoxia signaling in the pathogenesis of pulmonary fibrosis (Jain and Sznajder 2005; Tzouvelekis et al. 2007; Rabbani et al. 2010; Kottmann et al. 2012; Bodempudi et al. 2014). It is possible that a pathological loop exists in the fibrotic lung, in which hypoxia promotes fibroblasts proliferation and inflammatory damages, which in turn worsen hypoxia. It would be very interesting to further study how increased O_2_ saturation could benefit pulmonary injury, inflammation, or fibrosis.

In summary, this study supports further clinical evaluation of allosteric Hb modifiers as a novel therapeutic strategy for treating hypoxemia associated with pulmonary fibrosis and potentially retarding the progression of pulmonary fibrosis. GBT440, an analog of GBT1118 that increases Hb–O_2_ affinity and arterial O_2_ loading as well, is currently in clinical trials for the treatment of sickle cell disease and has demonstrated excellent safety and biological effects in both healthy subjects and subjects with sickle cell disease (NCT02285088, http://clinicaltrials.gov/) (Oksenberg et al. 2016).

## Conflict of Interest

All authors of this manuscript are paid employees and stockholders of Global Blood Therapeutics.
